# A simple workflow to identify novel small linear motif (SLiM)-mediated interactions with AlphaFold

**DOI:** 10.1093/bib/bbaf501

**Published:** 2025-09-28

**Authors:** Martin Veinstein, Victor Janssens, Bogdan I Iorga, Raphaël Helaers, Thomas Michiels, Frederic Sorgeloos

**Affiliations:** Université Catholique de Louvain, de Duve Institute, Brussels, Belgium; Université Catholique de Louvain, de Duve Institute, Brussels, Belgium; Université Paris-Saclay, CNRS, Institut de Chimie des Substances Naturelles (ICSN) , Gif-sur-Yvette, France; Université Catholique de Louvain, de Duve Institute, Brussels, Belgium; Université Catholique de Louvain, de Duve Institute, Brussels, Belgium; INRS-Centre Armand-Frappier Santé Biotechnologie, Laval, Québec, Canada

**Keywords:** small linear motif, SLiM, AlphaFold, intrinsically disordered protein, protein–protein interactions, RSK, p90 ribosomal S6 kinase

## Abstract

Short linear motifs (SLiMs) are highly compact interaction modules embedded within disordered protein regions and are increasingly recognized for their central role in maintaining cellular homeostasis. Due to their small size, degeneracy and transient binding, SLiMs remain difficult to detect both experimentally and computationally. Here, we show that AlphaFold (AF), used via ColabFold, offers a practical and accessible alternative for *in-silico* screening of new SLiMs targeting a protein of interest. Unlike previous studies that evaluated AlphaFold2 (AF2) using structure-derived benchmarks, we extend this by assessing both AF2 and AF3, using a structure-independent benchmark of 26 interactions absent from PDB homology, and showing that MiniPAE is the most suited AlphaFold metric for SLiM screening. We also generated an unbalanced dataset with a large excess of non-binders mimicking real-world blind screening, revealing a critical limitation in AlphaFold’s specificity for SLiM detection. To circumvent this constraint, we propose both a SLiM screening strategy and an adaptative scoring threshold. For greater accessibility, we provide a streamlined and cost-effective AF analysis workflow requiring no local installation or computation. To overcome challenges associated with SLiM validation, we also introduce a highly sensitive detection method based on proximity labeling in living cells. This workflow was used to identify and experimentally validate 13 new SLiMs that mediate binding to ribosomal protein S6 kinase A3 (RPS6KA3 or RSK2). By leveraging ColabFold and MiniPAE available through Colab notebooks, our approach provides a scalable and widely accessible strategy for identifying functional SLiMs in proteins of interest. MiniPAE can be accessed at https://github.com/martinovein/MiniPAE

## Introduction

Short linear motifs (SLiMs) are short regions of 3–10 amino acids embedded within intrinsically disordered regions (IDRs) that mediate protein–protein interactions (PPIs). These interactions generally occur between a SLiM and a structured protein domain and exhibit low-to-mid micromolar affinities, making them well suited for dynamic and reversible interactions that regulate cell signaling [1]. SLiMs control a wide range of biological processes and are catalogued in resources such as the eukaryotic linear motif (ELM) database, which currently contains around 1500 annotated human SLiMs [2]. However, given the estimated tens of thousands of human SLiMs, the vast majority of them remain undiscovered [1].

Despite their importance, SLiMs are notoriously challenging to detect directly due to their small size and weak binding affinities. Alternative *in-vitro* methods have been developed to improve detection sensitivity [3]. However, these approaches remain labor-intensive, difficult to scale-up and still face intrinsic affinity limitations. As such, computational approaches have emerged as powerful complementary tools to accelerate SLiM discovery.

Despite not being specifically designed for this purpose, AlphaFold2 (AF2) has shown an unexpected ability to model SLiM-mediated interactions. An early study demonstrated that AF2 could accurately predict the structure of interactions between IDRs and their structured binding domains, even for protein pairs entirely absent from its training set [4].

Subsequent benchmarking by Lee *et al.* provided a systematic comparison of different AAF-based metrics for SLiM–domain interactions [5]. Notably, they were among the first to apply AF at scale to screen for SLiM-mediated PPIs across an entire signaling network. While their results highlighted the limited specificity of AF in this context, they did not propose a strategy to address this limitation.

More recently, Omidi *et al.* conducted a comprehensive analysis of AF2’s ability to model a larger variety of IDR-mediated interactions including SLiMs, molecular recognition features (MoRFs), among others [6]. Importantly, they introduced two new residue-level metrics: MinD, which evaluates the minimum distance between residues across interfaces, and MiniPAE, derived from AF’s predicted aligned error (PAE) maps, to identify potential interaction regions within full-length IDRs.

In this study, we build on previous evaluation of AF2 for SLiM discovery by extending the comparison to AF3 [7] and introducing a structure-independent benchmark of 26 interactions lacking PDB homology. Using a large, unbalanced dataset mimicking real-world blind screening, we expose a critical limitation in AF’s specificity. To address this, we propose a new SLiM screening strategy that incorporates an adaptive scoring threshold to select high-confidence predictions. This analysis led to a streamlined and accessible workflow for SLiM discovery, which we have made available through a user-friendly Colab notebook. We illustrated the power of this approach with a case study focused on identifying RSK-binding SLiMs. To complement our *in-silico* predictions and address the challenge of low-affinity interactions, we further introduced a sensitive proximity labeling method, enabling functional validation, in cells, of candidate SLiMs predicted by our pipeline.

## Results

### Benchmark SLiM datasets

**Figure 1:**
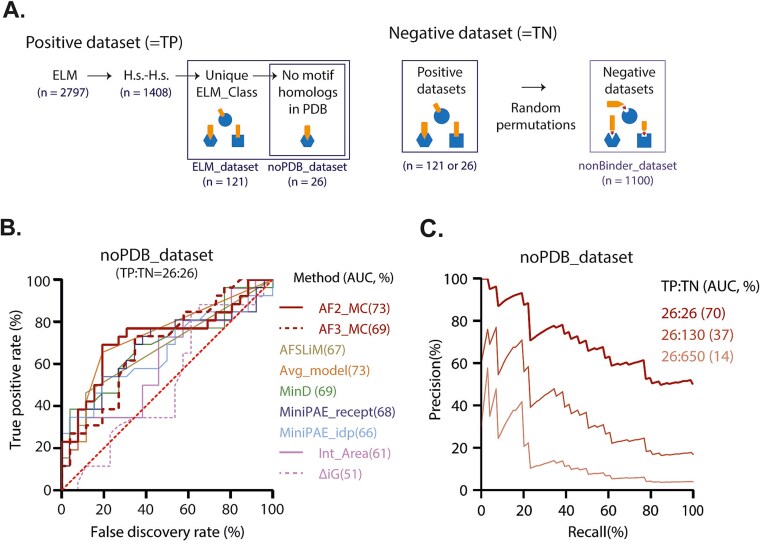
Benchmarking of AlphaFold-based metrics for SLiM-mediated interaction detection. (A) Construction of benchmark datasets. A non-redundant set of 121 human SLiM–partner pairs, each representing a unique ELM_Class, was extracted from the ELM database resulting in the ELM_dataset (Supplementary data S1). A subset of 26 instances lacking structural homologs in the protein data bank (PDB) constituted the noPDB_dataset. Negative datasets were generated by randomly permuting the interaction partners within each positive dataset (see method). (B) Receiver operating characteristic (ROC) curves comparing true-binders to randomly shuffled non-binders, using the balanced noPDB_dataset. Seven scoring metrics are compared: Model confidence (MC), AlphaSLiM (AFSLiM), average_model from Schmid *et al*. [9] (Avg_model), the minD and miniPAE metrics from Omidi *et al*. [6], interface area, and solvation free energy (ΔiG). Area under the curve (AUC) values are indicated between parentheses for each metric. AF2 = AlphaFold2, AF3 = AlphaFold3, TP = true positives, and TN = true negatives. (C) Precision-recall curves evaluating AlphaFold2 Model confidence ability to distinguish binders from non-binders at different binder to non-binders ratios.

To assess AF’s capacity to identify SLiMs within binary interaction predictions, we constructed a benchmark dataset derived from the ELM database, a manually curated repository of experimentally validated SLiMs. Given the high redundancy of SLiMs, the ELM database groups analogous motifs under a common classification, termed ELM_Class. Among the 161 ELM_Classes annotated in humans, we selected 121 representative instances, each corresponding to a distinct ELM_Class, to serve as our positive dataset, hereafter referred to as the ELM_dataset (see Methods, Fig. 1A, Supplementary data S1).

Unlike structure-centric benchmarks, our evaluation does not require the existence of homologous complexes in the protein data bank (PDB). This allowed us to form a subdataset of 26 SLiM–partner pairs which have no available structural homologs. This dataset was named noPDB_dataset (Fig. 1A, Supplementary data S1).

To rigorously evaluate AF’s predictive performance in different screening scenarios, we generated a large negative control set by randomly shuffling the binding partners from our positive dataset, following the strategy of Lee *et al.* [5] (Fig. 1B, Supplementary Data S1). From all possible shuffled pairs, 1100 likely non-binding combinations were randomly selected to serve as ‘non-binders’ (see Methods).

### Comparison of different AF2/3 metrics to discriminate SLiM-binders from non-binders

AF’s utility in SLiM discovery can be conceptually divided into two challenges. The first is binary ‘classification’—determining whether a SLiM mediates binding to a partner at all. The second is ‘localization’—identifying the specific region that functions as a SLiM. For those tasks different AF-based metrics can be used.

Among those, pLDDT has garnered considerable attention as a metric for localizing SLiMs [8]. However, it suffers from a fundamental limitation: its scores tend to increase not only where a disordered region folds upon binding (the signal of interest), but also in pre-structured regions that are not involved in the interaction (noise). To address this limitation, we developed AlphaSLiM, a new scoring metric that quantifies the change in per-residue pLDDT between the unbound and bound states of an IDR (see Methods). We compared AlphaSLiM to four other AF-based metrics: the model confidence (MC) computed by AF, the average_model proposed by Schmid *et al.* [9], and the minD and miniPAE scores proposed by Omidi *et al.* [6]*.*

Using our ELM_dataset, we first evaluated these metrics for their ability to distinguish true SLiM-binders from non-binders. All metrics—AlphaSLiM, MC, average_model, minD, and miniPAE—achieved comparable performance, with ROC area under the curve (AUC) values around 70% (±3%) (Supplementary Fig. S1A, Supplementary data S1) suggesting that AF-based scoring functions can moderately discriminate binders from non-binders. Importantly, when we repeated the evaluation using the noPDB_dataset, results remained consistent (AUC = 70% ± 4%, Fig. 1B, Supplementary data S1), indicating that the observed performance of AF-based metrics in distinguishing binders from non-binders did not arise from structural biases linked to PDB templates.

To assess AF’s performance under real world SLiM screening conditions in which non-binders vastly outnumber true binders, we leveraged our large nonBinder_dataset to generate precision-recall curves using increasingly unbalanced datasets (1:1, 1:5, and 1:25 binder to non-binder ratios). As shown in Fig. 1C, AF’s performance dropped sharply as the ratio became more unbalanced. The AUC fell from ~70% in a balanced setting (1:1) to 37% and 14% at 1:5 and 1:25 ratios, respectively. These results highlight AF’s limited specificity in blind screening contexts when true binders are rare (e.g. 1:1000).

To evaluate whether newer model architectures improve SLiM detection, we compared AF2 and AF3. To avoid biases stemming from differences in training data, especially with respect to known structural templates, we restricted this comparison to the noPDB_dataset. Our results revealed comparable performance across AF2 and AlphaFold3 metrics in terms of binder versus non-binder discrimination, suggesting that despite its architectural advances, AF3 MC does not provide a clear advantage for SLiM binders from non-binders discrimination (Fig. 1B, Supplementary data S1).

### Comparison of different AF2/3 metrics to identify new SLiMs within full-length proteins

**Figure 2:**
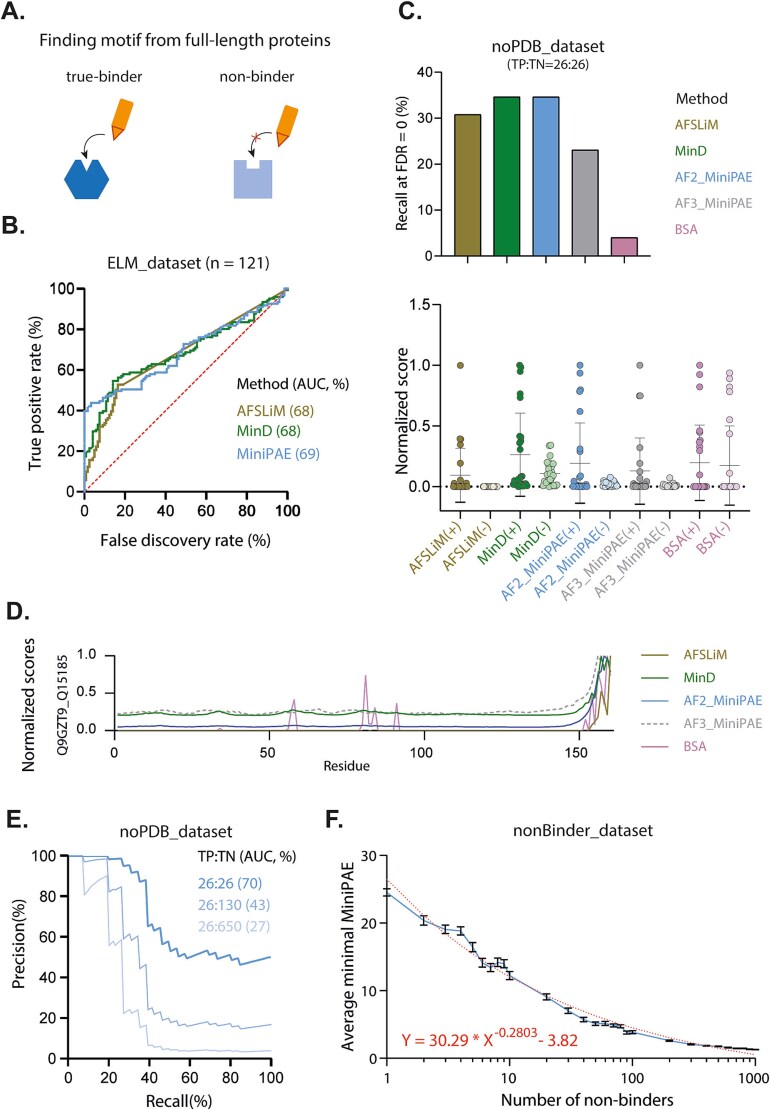
Using AlphaFold metrics to localize SLiMs within full-length proteins. (A) Schematic of the analysis: Scores within the motif region when paired with its true-binder are compared to the score within the same region when paired with a random non-binder. (B) ROC curves for the ELM_dataset. (C) Top: True positive rate (TPR, %) at FDR = 0. Bottom: Distribution of scores in the motif region for true-binders (+) versus non-binders (−) on a 0,1 scale (see method). BSA = buried surface area. (D) Example of per-residue score profile. (E) Precision-recall curves evaluating AlphaFold2 MiniPAE ability to identify SLiM with full-length proteins in unbalanced scenarios. TP:TN indicates binder-to-non-binder ratios. (F) Distribution of the average minimal value for MiniPAE score with increasing numbers of non-binders (see method). A power-law curve was fitted to model the relationship between dataset size and optimal threshold.

We next assessed AF’s ability to localize a binding SLiM within full-length proteins. For this, we compared the per-residue score within the SLiM region when paired with its true partner to the score within the same region when paired with a random non-binder (Fig. 2A). With our ELM_dataset, all three AF-derived metrics showed similar overall performances (AUC ~78%, Fig. 2B), but miniPAE outperformed the others at FDR = 0. This advantage disappeared with the noPDB_dataset (Fig. 2C and D; Supplementary Fig. S1B), suggesting that miniPAE’s precision is driven by structural similarity to known templates. Of note, physics-based metric like the buried surface area (BSA) metric performed significantly worse, with recall at FDR = 0% falling from ~30% to 4% (Fig. 2B).

The large overlap between true/false motif calls from AlphaSLiM, minD, and miniPAE (Supplementary Fig. S1C) suggests that combining these metrics offers little added benefit. Additionally, although shorter prediction lengths are known to improve structural accuracy [4, 5], we found no correlation between prediction length and motif detection (Spearman ρ = 0.03 and −0.17 for ELM_dataset and noPDB_dataset, respectively; Supplementary Fig. S1D-E).

Since both AF2 and AF3 output PAE scores, we used the noPDB_dataset to compare their ability to localize SLiMs within proteins. AF3 showed slightly lower performance than AF2 in this task (Fig. 2C, Supplementary Fig. S1B; Supplementary data S1).

Figure 1C highlighted a sharp drop in AF2 performance under unbalanced conditions where non-binders greatly outnumber binders, a common scenario in SLiM screening. To explore this further, we leveraged our larger nonBinder_dataset (*n* = 1100) to evaluate, using precision-recall curves, the ability of AF2’s MiniPAE metric to identify SLiMs within full-length proteins in increasingly unbalanced scenarios. Figure 2E confirmed a marked decline in performance as the binder-to-non-binder ratio increased, with AUC values dropping from 70% (1:1) to 43% (1:5) and 27% (1:25).

To address this limitation, we analyzed how the distribution of MiniPAE scores shifts with increasing numbers of non-binders. This analysis, presented in Fig. 2F, allowed us to derive an adaptive thresholding scheme by fitting a power-law function to the data. This formula enables dynamic adjustment of the scoring threshold based on the scale of the screening campaign: when screening hundreds of potential partners, a stringent threshold minimizes false positives, while in focused analyses involving a known interactor, a less stringent threshold maximizes recovery.

### AF-based workflow to identify new SLiMs binding a protein of interest

**Figure 3:**
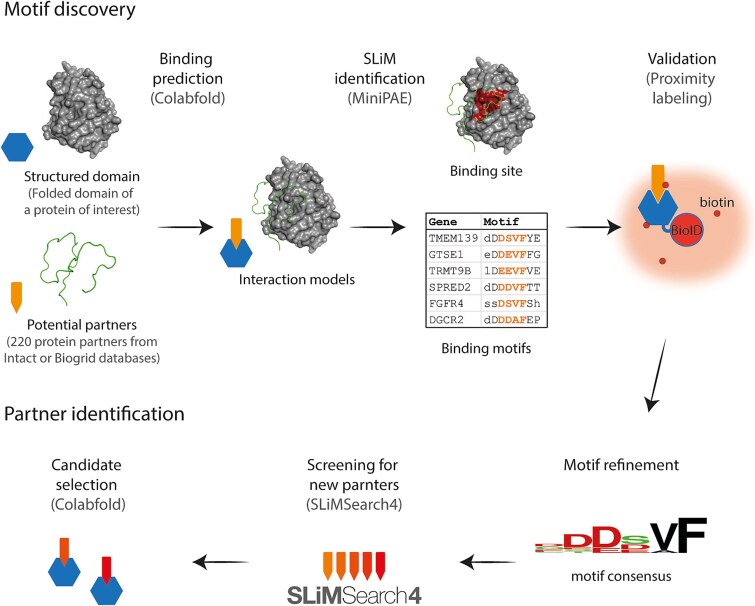
An AlphaFold-based workflow to identify new SLiMs. ColabFold is used to perform binary binding predictions between a protein (or domain) of interest and selected candidates from interaction databases or in-house datasets. The MiniPAE script extracts high-confidence SLiM predictions, which can be experimentally validated using proximity labeling strategy owing to their typically low affinity. Finally, this motif knowledge can be used to discover new interactants of your protein of interest using SLiMSearch4. All *in-silico* tools can be accessed via Colab notebooks or webservers: ColabFold: https://colab.research.google.com/github/sokrypton/ColabFold/blob/main/AlphaFold2.ipynb#scrollTo=kOblAo-xetgx. AF2_MiniPAE: https://colab.research.google.com/drive/1ZAj5gQOP-usd8yAK5uhoLsLH4WPAKxMG. SLiMsearch4: https://slim.icr.ac.uk/tools/slimsearch/input.

Building on our benchmarking results, we propose a step-by-step workflow to discover new SLiMs binding a specific protein (Fig. 3), while highlighting common pitfalls in such AF screenings. The first critical decision lies in selecting candidate protein partners for binary prediction. An overambitious approach would be to perform blind screening against entire proteomes. In line with this, Figs 1C and 2E demonstrate that AF-based metrics perform poorly when non-binders vastly outnumber true-binders, a behavior also reported by Schmid *et al.* [9]. To mitigate this, we recommend selecting partners from curated interaction databases such as BioGRID [10] or IntAct [11], or from in-house datasets. Given that AF2 slightly outperforms AF3, and is readily accessible, we suggest its use for most cases.

To streamline SLiM retrieval, we adapted the MiniPAE script from Omidi *et al.* [6] to analyze AF2 output files, returning MiniPAE scores, motif sequences, and coordinates. Since experimental validation is often more limiting than prediction time, a stringent threshold is recommended to prioritize high-confidence candidates. In line, an adaptive MiniPAE threshold was defined in Fig. 2F as:


$$ \mathrm{MiniPAE}\ \mathrm{threshold}=30.29\times{n}^{-0.2803}-3.82 $$

where *n* represents the number of predictions analysed.

With regard to SLiMs, validating them remains a significant challenge given the low affinity interaction that they often mediate [3]. To mitigate this, we propose a proximity labeling assay as a low-cost, sensitive method to detect interactions in living cells. In this approach, the predicted SLiM was expressed as a fusion protein (see Methods), and proximity labeling was achieved by fusing the target protein to BioID2, a promiscuous biotin ligase that covalently biotinylates proteins within ~10 nm in living cells [12]. If the SLiM mediates an interaction with the target, the SLiM fusion protein is expected to undergo increased biotinylation, reflecting its proximity to the BioID2-tagged target. Once validated, interacting motifs can be aligned to infer the minimal binding consensus. While identifying the interaction motif of a known partner is informative, the ultimate goal is often to discover new binders. This can be achieved using tools such as SLiMSearch4 [13], which scans the proteomes for additional motif matches. Depending on the specificity of the consensus, this may yield a narrow or broad spectrum of candidates that can be further refined using AF to evaluate whether the motifs adopt a compatible binding interface *in-silico*.

### Illustration of the use of our workflow to find hRSK2-binding SLiMs

To illustrate the practical application of our SLiM discovery workflow, we used it to identify novel motifs binding human RSK2 (hRSK2), a Ser/Thr kinase broadly expressed across tissues and involved in numerous biological processes [14, 15]. Mutations in RSK2 are known to cause Coffin-Lowry syndrome [16] and have been implicated in several other disorders [14], underscoring the importance of understanding how RSK2 selectively engages its partners through SLiMs.

As outlined in the previous section (Fig. 3), we started by selecting candidate RSK2-binding proteins. For this, we queried the BioGRID and IntAct interaction databases, and, recognizing RSK2’s role as a kinase, we also included all known substrates listed for RSK2 in PhosphoSitePlus [17]. This resulted in a list of 220 proteins with less than 1000 amino acids (longer proteins were not considered), which were then individually modeled in binary complexes with RSK2 using AF2. The resulting structures were analyzed using our adapted MiniPAE script, and motifs were filtered using a MiniPAE threshold of 2.85, calculated based on the equation from the previous section, with *n* = 220. This analysis yielded 18 predicted SLiMs from 17 proteins.

**Figure 4:**
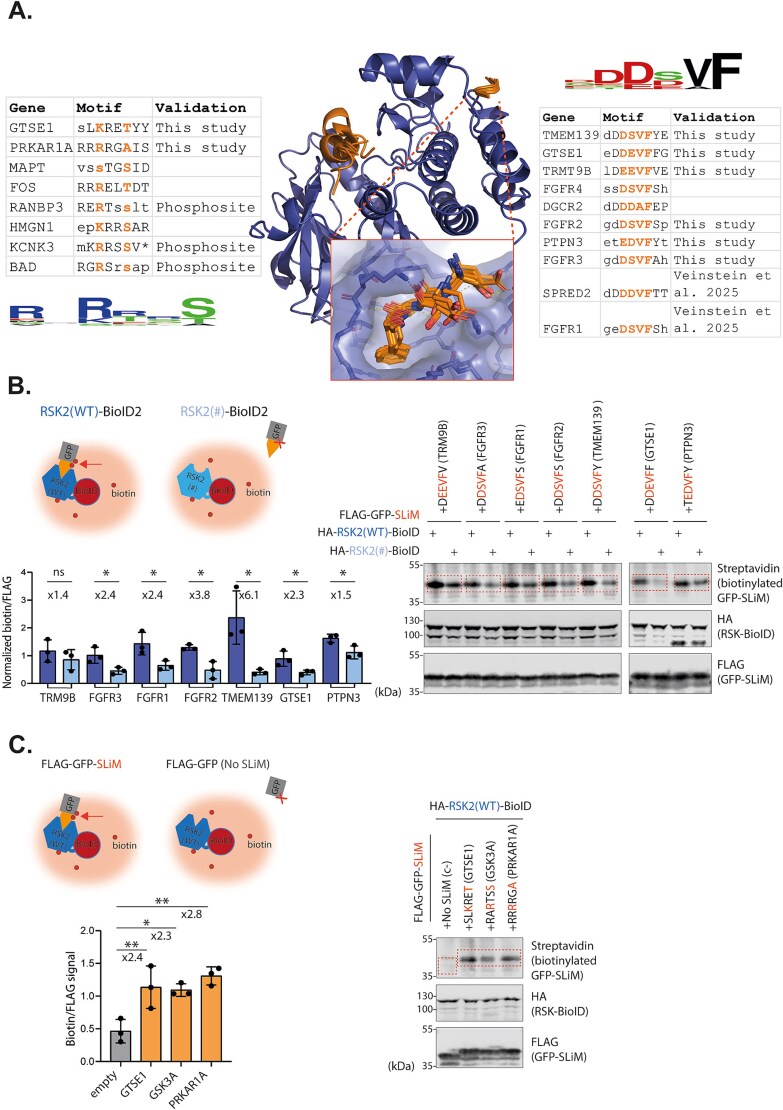
Discovery and validation of hRSK2-binding SLiMs using our AlphaFold-based workflow. (A) AlphaFold2 predictions show two classes of SLiMs binding to hRSK2: Substrate motifs (left) and DDVF-like motifs (right), along with their associated consensus logos. (B–C) Experimental validation of SLiM–RSK2 proximity using a BioID2 proximity labeling. Fold changes and statistical significance are indicated; comparison bars show the groups being tested. Ns = not significant, ^*^ = *P* < 0.05, ^**^ = *P* < 0.01. B: DDVF-like motifs were tested with wild-type or KSEPPY-mutant RSK2. Statistical analysis was performed using a two-tailed unpaired t-test. C: Substrate motifs were tested with wild-type RSK2 and compared to the empty construct. Statistical analysis was performed using one-way analysis of variance (ANOVA). Schematics (top), representative immunoblots (right), and quantifications (left) confirm motif-specific proximity labelling (n = 3).

Predicted SLiMs clustered into two distinct binding classes. The first group consisted of 10 ‘DDVF-like’ motifs that engaged a region centered on the KAKLGM loop of RSK2, a surface previously implicated in regulatory interactions and viral hijacking [18, 19]. The second group, comprising 8 ‘substrate motifs’, bound the catalytic cleft of RSK2 and resembled canonical substrate motifs (Fig. 4A, Supplementary data S2).

To experimentally validate these predictions, we selected a subset of representative SLiMs from each class. Among the DDVF-like motifs, two had previously been shown to bind RSK1, consistent with the fact that the closely related RSK1 and RSK2 share the same KAKLGM loop [18, 19]. We focused on six new DDVF-like motifs for validation. Given the low affinity of SLiM interactions, we used a proximity labeling strategy involving BioID2 fused to RSK2. Each predicted SLiM was cloned at the C-terminus of a FLAG-GFP construct (see Method), and biotinylation of this protein was compared between cells expressing the wild-type RSK2 or a KAKLGM-to-KSEPPY mutant previously shown to reduce DDVF-mediated interactions. For five out of six SLiMs examined, the KAKLGM-to-KSEPPY mutation in RSK2 significantly reduced biotinylation of the FLAG-GFP-SLiM constructs, confirming RSK2 KAKLGM loop-dependent interactions (Fig. 4B). The degree of biotinylation enrichment by wild-type versus mutant RSK2 varied across SLiMs, ranging from 1.5-fold for PTPN3 to 6.1-fold for TMEM139. Although TRM9B showed a 1.4-fold increase in biotinylation by wild-type RSK2, this difference was not statistically significant at the *P* < 0.05 level.

For the ‘substrate SLiMs’, five of the eleven predicted motifs were already annotated as RSK2 phosphorylation sites according to PhosphositePlus. We selected two uncharacterized motifs from the remaining six. Validating these was more challenging: substrate motifs are not expected to form stable interactions with kinases due to the transient nature of catalysis, and there are no well-characterized mutations that abolish substrate ‘binding’ without affecting kinase function. Therefore, we assessed proximity by comparing the biotinylation levels of FLAG-GFP-SLiM constructs to those of FLAG-GFP alone. As shown in Fig. 4C, the addition of the motif led to a twofold increased biotinylation, suggesting that our predicted substrate motifs were indeed contributing to the interaction with RSK2. Notably, these motifs matched the known consensus sequence for RSK2 substrates, as curated in PhosphoSitePlus, reinforcing the validity of our predictions.

**Figure 5:**
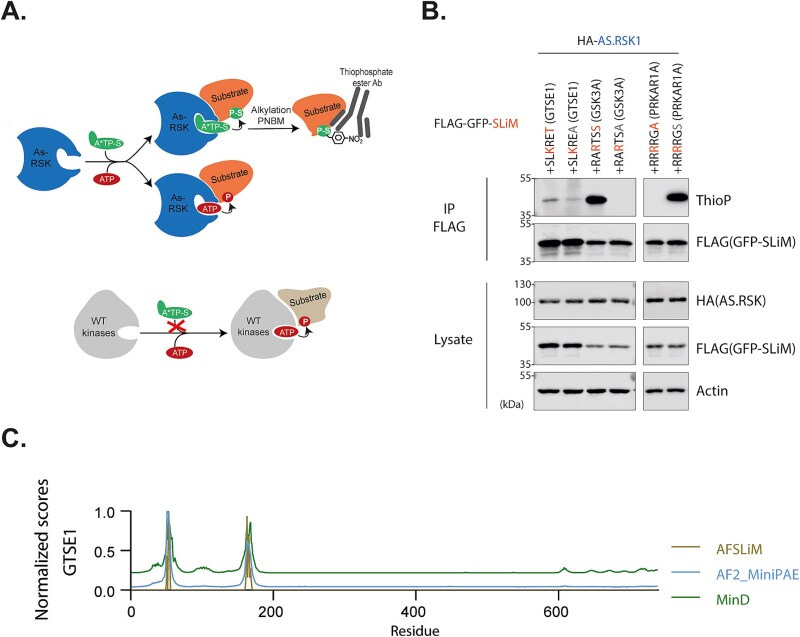
RSK1-dependent phosphorylation of predicted substrate SLiMs using an RSK1 analog-sensitive kinase. (A) Schematic representation of the analog-sensitive kinase system (adapted from Lizcano-Perret *et al*. [20]. The analog-sensitive (AS) kinase transfers a thiophosphate group from N^6^-bn-ATP-γ-S (a^*^TP-S) to its substrates, enabling subsequent alkylation with PNBM and detection using an anti-thiophosphate ester-specific antibody. (B) Representative immunoblots showing thiophosphorylation of immunoprecipitated FLAG-GFP-SLiM proteins harboring predicted substrate motifs. Constructs were transfected in Hela cells expressing HA-tagged AS-RSK1. Wild-type motifs exhibit greater RSK1-dependent thiophosphorylation compared to their mutant counterparts (n = 2). (C) Different AlphaFold-based scores along GTSE1 sequence when paired with RSK2.

While these results showed that substrate motifs conferred proximity to RSK2, this alone could not confirm whether these were actually phosphorylated by RSK. To address this, we used an analog-sensitive (AS) kinase system previously developed for RSK1 by Lizcano-Perret *et al*. [20], taking advantage of the high sequence conservation across RSK paralogs [15]. This system enabled the specific detection of phosphorylation events mediated by an analog sensitive RSK (Fig. 5A). For both GTSE1 and GSK3A substrate motifs, phosphorylation of the wild-type SLiM was significantly higher than that of the non-phosphorylatable serine-to-alanine (S > A) mutant, confirming RSK2-dependent phosphorylation (Fig. 5B). Notably, the phosphorylation signal for GTSE1 was markedly weaker than for GSK3A, which could be explained by the fact GSK3A has a canonical substrate motif while GTSE1 does not [15]. We hypothesize that GTSE1’s dual DDVF and substrate motifs may compensate for its non-canonical sequence, facilitating phosphorylation in the context of the full-length protein, an hypothesis supported by the work of Alexa et al., on analogous motifs [21]. In the case of a predicted pseudosubstrate motif in PRKAR1A that naturally contains a non-phosphorylatable alanine at the phospho-acceptor site, we did the converse and introduced an alanine-to-serine mutation and observed phosphorylation of this mutant, further supporting the accuracy of our prediction.

Altogether, our experimental setup allowed to confirm, to various degrees, seven out of eight tested SLiMs, supporting the use of an adaptative MiniPAE threshold for predictive screening. Regarding substrate motifs, our results suggest that AF predictions can extend beyond structural binding to inform on enzymatic modification events, consistent with previous suggestions by Lee *et al*. [5] in the context of protease cleavage site prediction.

## Material and methods

### Benchmark

We downloaded all 2797 interaction instances from the ELM database and retained only those corresponding to *Homo sapiens–H. sapiens* interactions. Following the filtering strategy of Bret *et al.* [4], we excluded instances in which the same motif occurred multiple times within a single protein, as this can complicate result interpretations. We also removed a small number of entries for which the reported motif consensus (‘regular expression’) could not be located within the corresponding protein sequence. To avoid redundancy, we kept the shortest instance per ELM class. In addition, to reduce prediction time, we restricted both motif-containing proteins and their partners to sequences shorter than 1000 residues, yielding a final benchmark set of 121 interactions—referred to as the ‘ELM_dataset’ (see Fig. 1A)—across 161 unique human ELM classes.

Among these, only 26 interactions had no corresponding structural information available in the PDB according to ELM annotations; this subset is referred to as the ‘noPDB_dataset’.

As a negative control, we generated a ‘nonBinder_dataset’ by randomly shuffling SLiM–target combinations from the ELM_dataset, ensuring that each new pair was absent from the original ELM data. To avoid introducing functional motifs by chance, we excluded pairs in which the SLiM matched any regular expression associated with the new target. Finally, we retained only pairs with a total sequence length under 1000 residues and randomly selected 1100 such pairs as likely non-binders. All instances are provided in Supplementary data S1.

### AF prediction

Protein complex structures were predicted using AF2 via ColabFold in multimer V3 mode with default parameters. Predictions using AF3 were performed using the AF Server available at https://alphafoldserver.com/.

### MiniPAE and other scoring metrics

#### Regular AF-based metrics

Per-residue pLDDT and PAE matrices were extracted from ColabFold output files. Global confidence metrics (pTM, ipTM) were combined into a MC score: $MC=0.2\cdot pTM+0.8\cdot ipTM$ [5]

#### MiniPAE

For binary complex predictions, AF computes a PAE matrix that quantifies the confidence in the relative positioning of different residues. MiniPAE, introduced by Omidi *et al*. [6], represents the lowest (i.e. best) inter-protein PAE value in this matrix. We implemented a simplified MiniPAE script for both AF2 https://github.com/martinovein/AF2_MiniPAE and AF3 https://github.com/martinovein/AF3_MiniPAE and is available as a pseudocode in Supplementary Data S3.

#### AlphaSLiM score

While pLDDT is commonly employed for SLiM detection, its utility is limited by its increased values in both structured regions and disordered regions upon binding. To overcome this limitation, we developed the AlphaSLiM score, defined as:


$$ \mathrm{AlphaSLiM}\ \mathrm{score}=N\cdot \left(\mathrm{pLDDTbound}-\mathrm{pLDDTunbound}\right) $$

where *N* is the number interactions calculated by protein-ligand interaction profiler [22]. Motif regions were defined as contiguous residues with positive pLDDT differences, scored by their maximum AlphaSLiM score. While MiniPAE offers simpler implementation, AlphaSLiM remains available at https://github.com/martinovein/AlphaSLiM and as pseudocode in Supplementary Data S3.

We also used ‘MinD’, a metric described by Omidi *et al*. (accessible at https://github.com/alirezaomidi/AFminD), and the ‘Average_model score’, developed by Schmid *et al*. available at [9].

Local (per residue) and global (per complex) deltaG were calculated using PDBePISA webserver: https://www.ebi.ac.uk/pdbe/pisa/. Buried surface area (BSA) per residue and total interface area were also computed using the same tool.

To facilitate comparative visualization, all scores (AlphaSLiM, MiniPAE, MinD, and BSA) were normalized to a 0–1 scale when indicated. AlphaSLiM and BSA scores were normalized by division with the maximum observed value.


$$ {\mathrm{Score}}_{\mathrm{norm}}=\frac{\mathrm{Score}}{{\mathrm{Score}}_{\mathrm{max}}} $$

${\mathrm{Score}}_{\mathrm{norm}}$ = normalized score

${\mathrm{Score}}_{\mathrm{max}}$ = maximum score in the dataset

For MiniPAE and MinD, normalization was performed as follows:


$$ {\mathrm{Score}}_{\mathrm{norm}}=\frac{{\mathrm{Score}}_{\mathrm{in}\mathrm{v}}-{\mathrm{Score}}_{\mathrm{in}{\mathrm{v}}_{\mathrm{min}}}}{{\mathrm{Score}}_{\mathrm{in}{\mathrm{v}}_{\mathrm{max}}}} $$

${\mathrm{Score}}_{\mathrm{norm}}$ = normalized MiniPAE or MinD score

${\mathrm{Score}}_{\mathrm{inv}}=\frac{1}{\mathrm{Score}}$ = inverse of original score

${\mathrm{Score}}_{\mathrm{in}{\mathrm{v}}_{\mathrm{min}}}$ = minimum ${\mathrm{Score}}_{\mathrm{inv}}$ in the dataset

${\mathrm{Score}}_{\mathrm{in}{\mathrm{v}}_{\mathrm{max}}}$ = maximum ${\mathrm{Score}}_{\mathrm{inv}}$ in the dataset

### Fitting of power-law model on MiniPAE score

To characterize the relationship between the number of non-binders and the average minimum MiniPAE value, we performed 100 random subsamplings from our nonBinder_dataset (*n* = 1100). For each iteration, we calculated the minimum MiniPAE value and then averaged these minima across iterations. We fitted a power-law equation according to:


$$ y=a\cdot{x}^{-b}+c $$

where *x* is the number of non-binders, *y* is the corresponding average minimum MiniPAE value, and *a*, *b*, and *c* are model parameters. The fitting was performed in Python using the *curve_fit* function from the *scipy.optimize* module, which applies non-linear least squares optimization.

### Precision-recall curve analysis

Precision-recall (PR) curves and PR-AUC values were computed to assess AlphaFold scores performance on imbalanced data. Positive scores (*n* = 26) and negative scores (*n* = 1100) were extracted from the noPDB_dataset and nonBinder_dataset respectively. To simulate varying binder-to-non-binder ratios, we randomly sampled 26 (1:1), 130 (1:5), or 650 [1, 25] negative scores and combined them with the 26 positive scores. This process was repeated across 10 independent iterations per ratio, and precision-recall metrics were averaged. Precision and recall were defined as:


$$ \mathrm{Precision}=\mathrm{TP}/\left(\mathrm{TP}+\mathrm{FP}\right) $$

$$ \mathrm{Recall}=\mathrm{TP}/\left(\mathrm{TP}+\mathrm{FN}\right) $$

where TP, FP, and FN are true positives, false positives, and false negatives, respectively. PR curves were generated using scikit-learn’s precision_recall_curve, and PR-AUC was calculated by trapezoidal integration.

### RSK2 partner selection from BioGRID or IntAct

To identify candidate interaction partners, we searched for the protein of interest in the BioGRID and IntAct databases using its gene name (e.g. RPS6KA3 for RSK2). The resulting lists of interactors were filtered based on the following inclusion criteria: only human proteins with fewer than 1000 amino acids were retained. Duplicate entries were removed based on protein names and this curated list was then used for downstream analysis.

### Quantification and statistical analysis

ROC curves (Figs 1-2), t-test (Fig. 4B), and one-way ANOVA (Fig. 4C) were calculated as implemented in the Prism 8.0.02 statistical analysis software (GraphPad Software, Inc., San Diego, CA).

### Plasmids, retroviral and lentiviral constructs

Expression plasmids are presented in Supplementary data S4. Note that FLAG tagged proteins expressed by these vectors contain 3xFLAG tag which are referred to as FLAG- in the text and figures. pBLP10 and pMD108 plasmids expressing HA-RSK(WT)-BioID2 [23] and HA-RSK(mutant)-BioID2 were kindly provided by Belén Lizcano Perret and Melissa Drappier, respectively. BioID2 [12] coding sequence can be accessed through the Addgene collection (#74223).

All ‘FLAG-GFP-SLiM’ expression plasmids were created by inserting hybridized oligo between *Bsu*36I and *Xba*I restriction sites within pMV51 plasmid, a derivative of pTM952 [24, 25]. SLiMs coding sequences (Supplementary data S4) were generates by Novopro codon optimization tool (https://www.novoprolabs.com/tools/codon-optimization).

### Cell culture and transfection

HEK293T [26] and HeLa-BLP32 (expressing analog-sentive human RSK1 [20]) were maintained in Dulbecco’s Modified Eagle Medium (Lonza) supplemented with 10% fetal bovine serum (Sigma), 100 U/ml penicillin and 100 μg/ml streptomycin (Lonza). All cells were cultured at 37 °C in a humidified atmosphere containing 5% CO_2_.

Cells seeded the day before were transfected using Lipofectamine 2000 (ThermoFisher) according to the manufacturer’s instructions. For analog sensitive kinase experiments, cells were incubated with analog sensitive kinase buffer prior to lysis, following the protocol described by Lizcano-Perret *et al*. [23].

### Biotinylation experiment

Twenty-four hours post-transfection, the HEK293T cells were incubated with 5 μM biotin (diluted in culture medium) for an additional 24 hours at 37 °C. medium was subsequently removed and cells were lysed in Laemmli buffer.

### Immunoprecipitations and western blot analysis

Immunoprecipitation and Western blotting were done as previously described [18]. Briefly, transfected cells were lysed in lysis buffer (Tris–HCl 100 mM pH 8, NaCl 150 mM, NP40 0.5%, EDTA 2 mM, and supplemented with protease/phosphatase inhibitors (Pierce)) and centrifugated at 14,000 x g for 10 min at 4 °C. Cleared supernatants were incubated with anti-FLAG M2 Magnetic Beads (#M8823, Sigma-Aldrich) with gentle agitation for 4 hours at 4 °C. Magnetic beads were then washed three times with the lysis buffer. Immunoprecipitated proteins were detected by Western-blot analysis antibodies listed in Supplementary data S4.

## Discussion

Although SLiMs are thought to regulate nearly all biological processes, their detection remains notoriously challenging *in vitro* due to the transient and often low-affinity nature of their interactions. *In silico*, early sequence-based motif discovery tools (e.g. MEME, STREME) were considered ill-suited for SLiM prediction from large sequence databases because of the short and degenerate nature of these motifs [27].

However, recent advances in machine learning have changed this landscape. Recent tools such as AIUPred-binding [28] and MoRFchibi 2.0 [29] leverage disorder prediction and deep learning to identify potential SLiM regions with great speed and accuracy (Table 1). Remarkably, MoRFchibi 2.0 has been shown to outperform AF in identifying disordered interaction regions from full-length sequences [29]. Yet, a key limitation is that those approaches do not predict the SLiM’s binding partner nor provide structural insight into the interaction, which limits their applicability for screening SLiMs binding a specific target (Table 1).

**Table 1:** Comparison of different SLiM discovery methods.

	Method	Examples	Time	Cost	‘Receptor’ proteins to screen	‘SLiM’ proteins screened	Structural insight
In silico	Neural network sequence-based predictors	AIUPred-binding [27] or MorfChibi 2.0 (28)	Seconds	Free	na	Proteome-wide	no
	AlphaFold	Colabfold	Days	Free/+	1–10	1–1000	yes
In vitro	High throughput Phage display	ProPD [1]	Months	++	1–100	Proteome-wide	no
	Proximity labelling	BioID2	Weeks	+	1	1–100	no

These approaches are complementary to structure-based SLiM prediction methods: sequence-based tools enable rapid, large-scale scanning of disordered regions, whereas structure-based approaches like AF can pinpoint specific binding partners and generate structural hypotheses for SLiM–target interaction interfaces.

Our study extends previous evaluations of AF2 for SLiM recovery by including AlphaFold3 and leveraging a structure-independent, ELM-derived benchmark of 26 interactions entirely absent from PDB homology. This benchmark demonstrated that MiniPAE, computed on AF2 predictions, performs comparably to both MinD and our AlphaSLiM scoring function. While all three are effective, MiniPAE’s simpler implementation makes it particularly practical for general users, avoiding the large extra outputs of MinD and the need for additional monomer predictions required by AlphaSLiM.

Despite promising results, benchmarking revealed key limitations. In unbalanced datasets mimicking real-world screening—where non-binders vastly outnumber binders—precision decreases exponentially. This finding precludes blind SLiM screening with AlphaFold and instead suggests starting from interaction datasets already enriched for true-binders like those available on BioGRID and Intact. To mitigate false positives, we proposed adaptive score thresholds tailored to specific screening strategies. Alternatively, AF predictions could be combined with post-hoc rescoring tools such as SPOC [9], though current implementations are limited to human reference proteins.

Finally, users planning SLiM screening experiments should account for AF’s intrinsic constraints. The model performs less reliably for certain protein classes (e.g. coiled-coils, antibody–antigen complexes, and some viral proteins), and prediction time increases exponentially with sequence length.

MiniPAE scores derived from AF3 predictions were slightly lower than those from AF2, echoing recent observations in peptide–protein interaction benchmarking [30]. The transition from AF2’s transformer-based architecture to AF3’s diffusion-based generative model [7] may underlie this difference. These findings support AF2 as the more practical choice for this application, especially given current throughput limitations on the AF3 server.

From a biological perspective, the generalizability of this workflow across SLiM types appears promising. The workflow successfully identified two RSK2-binding SLiM classes in this work with distinct interaction properties—one mediating transient, substrate-like binding and the other forming more stable complexes—indicating its capability to detect diverse SLiM types. We identified 13 previously undescribed RSK2-binding SLiMs, and using our sensitive proximity labelling system, we confirmed 8/9 tested predictions experimentally. Among these, we found that GSTE1 interacts with RSK2 via two distinct SLiMs: a substrate-like motif divergent from the canonical phosphorylation site, and a DDVF motif, which likely engages a different region near the kinase active site. We hypothesized that this dual SLiM strategy may facilitate the phosphorylation of non-canonical substrates, expanding the functional repertoire of the kinase [21]. However, the functional relevance of these motifs remains to be confirmed in the context of full-length GTSE1, as our current evidence is limited to peptide-based assays.

High-throughput phage display screening of SLiMs has previously achieved a recall of 23% [1], but its elevated cost and substantial protein purification workload limit broader adoption (Table 1). In contrast, our workflow offers a comparable recall of 30% with minimal infrastructure requirements (Table 1). The rise of ColabFold has democratized the use of structure prediction, allowing any researcher to perform interaction predictions without the need for local installation. Building on this, we provide a Google Colab notebook for SLiM identification across multiple AF predictions—eliminating installation barriers entirely. Together, these tools aim to make AF-based SLiM prediction accessible even to researchers without coding expertise.

**Key Points**

- Various AlphaFold2/3 scoring metrics were systematically benchmarked for short linear motif (SLiM) detection using a unique dataset of 26 interactions with no PDB homology, expanding on previous AlphaFold2 evaluations.
- Given our experimental background, we highlight a critical and often-overlooked limitation, showing that AlphaFold lacks specificity when screening for rare SLiMs.
- A user-friendly and cost-effective AlphaFold-based workflow was specifically designed to identify novel SLiMs for a protein of interest.
- This computational workflow was paired with a sensitive *in-vitro* assay to offer a complete solution for low-affinity SLiM discovery and validation, allowing the identification of 13 previously uncharacterized SLiMs interacting with RSK kinases.
- Together, our workflow and associated validation assay offer an integrated pipeline for the discovery and validation of SLiM-mediated protein–protein interactions.

## Supplementary Material

Fig_S1_bbaf501

Supp_Data_S1_-_Benchmark_for_AlphaFold_SLiM_predictions_bbaf501

Supp_Data_S2_-_RSK2_SLiM_prediction_bbaf501

Supp_Data_S3_-_pseudocodes_bbaf501

Supp_Data_S4_-_plasmids_and_antibodies_bbaf501

## Data Availability

All data are available for download and biological material is freely available upon request. All benchmark Alphafold2 data (ELM_dataset including noPDB_dataset, ~200Gb in 5 parts) is available at: https://zenodo.org/uploads/15260141, https://zenodo.org/uploads/15260224, https://zenodo.org/uploads/15273252, https://zenodo.org/uploads/15273254, https://zenodo.org/uploads/15273263. The monomeric predictions additionally required for AlphaSLiM are available at: https://zenodo.org/uploads/15236595. AlphaFold3 predictions for noPDB_dataset are available at: https://zenodo.org/uploads/15236655. hRSK2 SLiM predictions are available at https://zenodo.org/uploads/15235709. The monomeric predictions required for AlphaSLiM are available at: https://doi.org/10.5281/zenodo.15236154
