# Supplementary figures and images for: A simple workflow to identify novel small linear motif (SLiM)-mediated interactions with AlphaFold
Source: Brief Bioinform. 2025 Sep 28;26(5):bbaf501. doi: 10.1093/bib/bbaf501 (PMC12476836; doi:10.1093/bib/bbaf501)

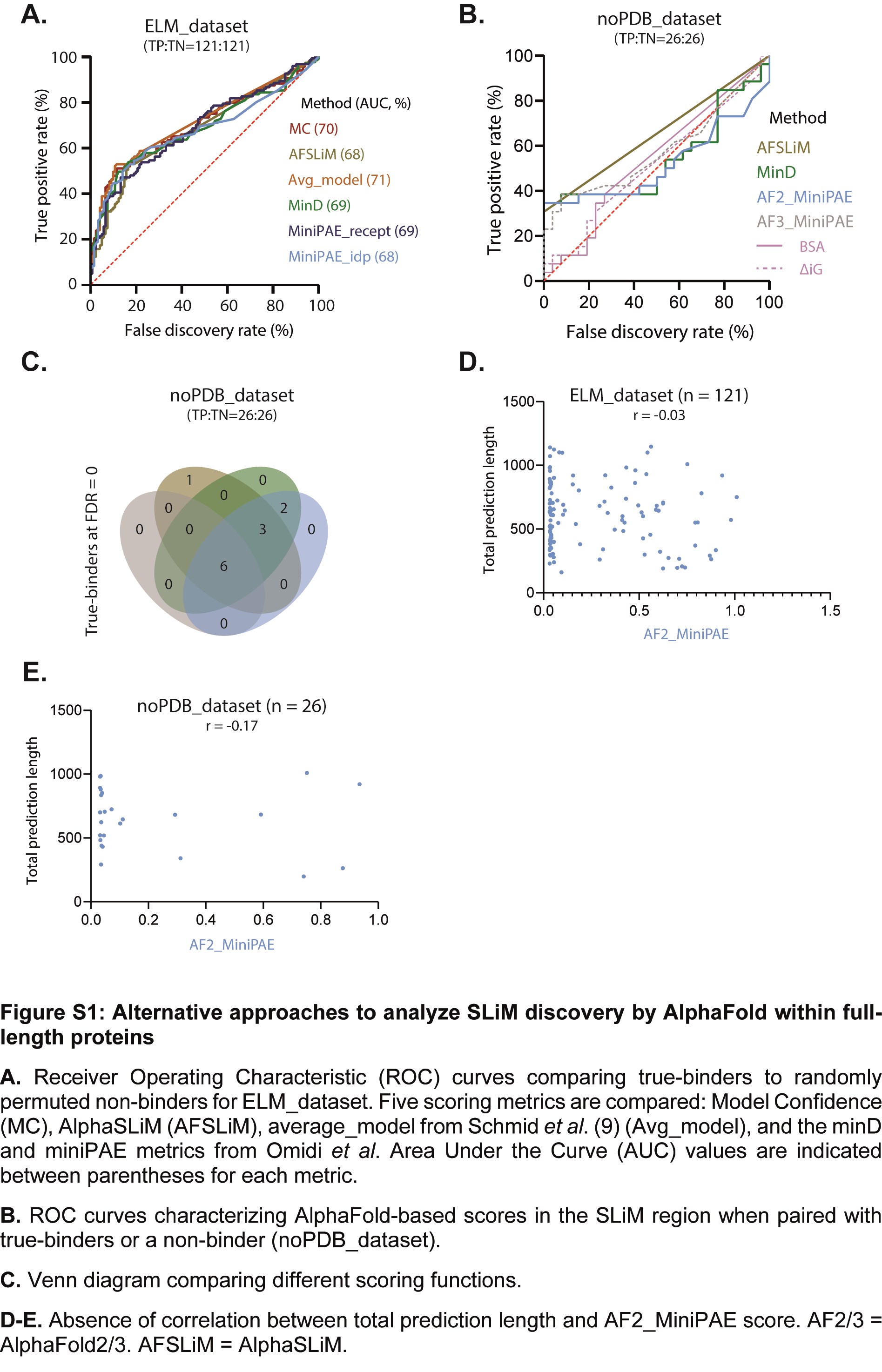

Supplement: Fig_S1_bbaf501 [file fig_s1_bbaf501.jpeg]
