# Supplementary material for: A simple workflow to identify novel small linear motif (SLiM)-mediated interactions with AlphaFold
Source: Brief Bioinform. 2025 Sep 28;26(5):bbaf501. doi: 10.1093/bib/bbaf501 (PMC12476836; doi:10.1093/bib/bbaf501)
Supplement: Supp_Data_S3_-_pseudocodes_bbaf501 [file supp_data_s3_-_pseudocodes_bbaf501.docx]

***MiniPAE pseudocode***

1. *Read alignment header (.a3m file) to extract SLiMProtein and DomainProtein lengths and sequences.*
2. *Load PAE matrix from scores_rank_001_.json.*
3. *Find the minimum PAE across all SLiMProtein residues.*
4. *Store the resulting list of miniPAE values for each SLiMProtein residue (miniPAE_list).*
5. *If a residue’s miniPAE < threshold → mark residue uppercase (high confidence).*
6. *Search for the longest contiguous uppercase segment = putative SLiM.*
7. *MiniPAE score = the lowest miniPAE value in the putative SLiM segment.*
8. *Record its sequence and 1-based start/stop positions.*
9. *Write results to CSV.*

***AlphaSLiM pseudocode***

1. *For each predicted complex (DomainProtein called ProtA, SLiMProtein called ProtB):*
   1. *Load AlphaFold rank_001 structure (.pdb)*
   2. *Run Protein-Ligand Interaction Profiler (PLIP) to identify interaction forces → save as XML.*
   3. *Convert PLIP XML report to JSON format.*
   4. *Extract pLDDT scores for SLiMProtein monomer and multimer .json files*
2. *For each ProtA_ProtB:*
   1. *Compute the pLDDT score difference between complex and monomer predictions.*
   2. *Multiply this value by the number of interactions as defined by PLIP*
   3. *Summarize results in an Excel file.*
3. *Merge all per-complex Excel files into a single mega Excel and CSV report.*
   1. *Motif regions are defined as contiguous residues with positive pLDDT differences, scored by their maximum AlphaSLiM value.*
